# Does mode of follow-up influence contraceptive use after medical abortion in a low-resource setting? Secondary outcome analysis of a non-inferiority randomized controlled trial

**DOI:** 10.1186/s12889-016-3726-1

**Published:** 2016-10-17

**Authors:** Mandira Paul, Sharad D. Iyengar, Birgitta Essén, Kristina Gemzell-Danielsson, Kirti Iyengar, Johan Bring, Marie Klingberg-Allvin

**Affiliations:** 1 Department of Women’s and Children’s health / International Maternal and Child Health (IMCH), Uppsala University, Akademiska Sjukhuset, Uppsala, SE-751 85 Uppsala Sweden; 2 Action Research & Training for Health (ARTH), 313011 Udaipur, Rajasthan India; 3 Department of Women’s and Children’s Health, Division of Obstetrics and Gynecology, Karolinska Institutet and Karolinska University Hospital, WHO collaborating Centre, SE-17176 Stockholm, Sweden; 4 Statisticon, SE-10136 Stockholm, Sweden; 5 School of Education, Health and Social Studies, Dalarna University, SE-791 88 Falun, Sweden

## Abstract

**Background:**

Post-abortion contraceptive use in India is low and the use of modern methods of contraception is rare, especially in rural areas. This study primarily compares contraceptive use among women whose abortion outcome was assessed in-clinic with women who assessed their abortion outcome at home, in a low-resource, primary health care setting. Moreover, it investigates how background characteristics and abortion service provision influences contraceptive use post-abortion.

**Methods:**

A randomized controlled, non-inferiority, trial (RCT) compared clinic follow-up with home-assessment of abortion outcome at 2 weeks post-abortion. Additionally, contraceptive-use at 3 months post-abortion was investigated through a cross-sectional follow-up interview with a largely urban sub-sample of women from the RCT. Women seeking abortion with a gestational age of up to 9 weeks and who agreed to a 2-week follow-up were included (*n* = 731). Women with known contraindications to medical abortions, Hb < 85 mg/l and aged below 18 were excluded. Data were collected between April 2013 and August 2014 in six primary health-care clinics in Rajasthan. A computerised random number generator created the randomisation sequence (1:1) in blocks of six. Contraceptive use was measured at 2 weeks among women successfully followed-up (*n* = 623) and 3 months in the sub-set of women who were included if they were recruited at one of the urban study sites, owned a phone and agreed to a 3-month follow-up (*n* = 114).

**Results:**

There were no differences between contraceptive use and continuation between study groups at 3 months (76 % clinic follow-up, 77 % home-assessment), however women in the clinic follow-up group were most likely to adopt a contraceptive method at 2 weeks (62 ± 12 %), while women in the home-assessment group were most likely to adopt a method after next menstruation (60 ± 13 %). Fifty-two per cent of women who initiated a method at 2 weeks chose the 3-month injection or the copper intrauterine device. Only 4 % of women preferred sterilization. Caste, educational attainment, or type of residence did not influence contraceptive use.

**Conclusions:**

Simplified follow-up after early medical abortion will not change women’s opportunities to access contraception in a low-resource setting, if contraceptive services are provided as intra-abortion services as early as on day one. Women’s postabortion contraceptive use at 3 months is unlikely to be affected by mode of followup after medical abortion, also in a low-resource setting. Clinical guidelines need to encourage intra-abortion contraception, offering the full spectrum of evidence-based methods, especially long-acting reversible methods.

**Trial registration:**

Clinicaltrials.gov NCT01827995

## Background

Family planning relies on simple technology and is the most cost-effective and feasible way to avoid unintended pregnancies [1]. Post-abortion contraception is an essential component of comprehensive abortion care [2] and provides a window of opportunity to motivate women to adopt contraception [3]. However, to successfully motivate women to initiate contraception they must be provided with appropriate counseling and a range of evidence-based contraceptive methods to choose from [4]. Allowing women to play an active role in deciding their preferred method and informing them about potential side effects and health outcomes can motivate adherence and continuation [5]. A study from Nepal shows that a majority (56 %) of women initiated a ‘modern’ or ‘traditional’ contraceptive method post-abortion, however, that discontinuation rates were high and half had discontinued within 12 months. Still, use of contraception was better and initiated earlier among post-abortion women compared with post-partum women [6].

There is a large unmet need for family planning (21 %) in India, especially among rural women. Women primarily rely on sterilization to limit the number of children [7], while they use ‘traditional methods’ to space between children [8, 9]. These findings can be explained by the persisting stigma attached to modern contraception use, especially in rural areas, resulting in young women’s fear of jeopardizing their social status if they were to use modern contraception [9]. An Indian national survey showed that 70 % of women who had an abortion did not use contraception post-abortion [10]. Conversely, in a study from two Indian states, Jharkhand and Bihar, the majority of women adopted contraception post-abortion when provided with comprehensive contraceptive counseling and a range of methods [11]. The National rural health mission (NRHM), the governmental effort to increase access to equitable health care, offers condom, oral pill, copper intrauterine device (IUD), and sterilization. Three-month injections, implants and hormone IUD are not covered by the government’s family planning scheme, however, injectables can be purchased in most hospitals or pharmacies [12] and there are ongoing discussions to whether or not the injectable should be included under the government family planning scheme. Abortion is legal and the national abortion guidelines advocate contraceptive counseling post-abortion and specify the suitable post-abortion contraceptive methods available [13]. In spite of this, less than half of women who received an abortion received post-abortion contraceptive counseling in Rajasthan and Maharashtra [14, 15].

Medical abortion is a non-surgical procedure similar to a spontaneous abortion and is induced by the combination of mifepristone, an anti-progesterone, and misoprostol, a prostaglandin E_1_ analogue [16, 17]. There is no clinical need for follow-up after a medical abortion [2], however, it is important to exclude the risk of ongoing pregnancies, and this can be resolved through the use of a home-based self-assessment regime [18, 19]. Published results from this randomized controlled trial (RCT) found that women’s home-assessment using a low-sensitivity pregnancy test (LSPT) and a pictorial instruction sheet 2 weeks after medical abortion is effective, acceptable and feasible [18, 20]. The efficacy of abortion among women who carried out home-assessment was non-inferior to those who attended in-clinic follow-up, and thus suggests the efficacy of simplified medical abortion in a low-resource and rural setting where women’s educational attainment is low [18]. However, simplified follow-up is not yet readily implemented and the practice of clinic follow-up after medical abortion persists in many settings. In India, women rarely return to the clinic for follow-up post-abortion, resulting in the health system’s lost opportunity to determine the outcome of the abortion as well as to provide post-abortion contraception [21]. While there are advantages in allowing women to confirm medical abortion outcome independently, without having to return to the clinic [18, 20], a potential disadvantage is that women will have decreased access to post-abortion contraceptive services. However, little is known about contraceptive use among women carrying out most of their medical abortion at home, especially in low-resource settings. This study examines whether there is evidence that such a disadvantage exists by comparing contraceptive use among women whose abortion outcome was assessed in-clinic with women who assessed their abortion outcome at home, in a low-resource, primary health care setting. Additionally, it investigates how background characteristics and abortion service provision influences contraceptive use post-abortion.

## Methods

### Trial design

This study was a randomized controlled non-inferiority trial with an additional 3-month cross-sectional follow-up interview with a sub-set of women from the RCT. The RCT compared clinic follow-up with home-assessment at 2 weeks after early medical abortion in a low-resource setting in Rajasthan, India. The 3-month follow-up was carried out to monitor the use and continuation of methods, and to determine whether different trends of contraceptive use could be established in the two study groups. The RCT followed CONSORT guideline format for non-inferiority randomised trials [22]. The primary outcome of the RCT was to measure the efficacy of home-assessment of women’s abortion outcome. Contraceptive use was measured as a secondary outcome of the RCT and the primary outcome of the cross-sectional sub-study. Details of the RCT implementation, setting and context, in addition to the methods, are described in the study protocol [23]. Results related to the efficacy of simplified follow-up after medical abortion are reported elsewhere [18].

### Study setting and participants

The RCT was conducted at six primary health care facilities; three clinics situated in an interior rural area in the Araveli mountain range, characterized by limited road connections, poverty, and strong gender disparities; and three clinics in Udaipur City. The rural clinics cater to women belonging to a lower socio-economic stratum and the area is largely populated by social groups that are referred to as marginalized [7] and defined by the government as scheduled castes (SC) and scheduled tribes (ST). The urban clinics cater mainly to urban women from all socio-economic strata. Women were eligible to participate in the study: when seeking medical abortion; with a pregnancy of up to 9 weeks of gestation, as estimated by pelvic examination; if they resided in an area where follow-up was possible or had a phone of their own; and if they agreed to a follow-up appointment 2 weeks after the abortion. For the 3-month follow-up, women were included if they: owned a phone; were recruited in the urban study sites; and agreed to a telephonic follow-up at 3 months. Women with known contraindications to medical abortion; who had a haemoglobin value below 85 mg/l; or who were below 18 years of age were excluded.

### Intervention and procedure

Before study recruitment, a doctor assessed all women seeking abortion and identified the women who were eligible, and who had opted for a medical abortion. These women were counselled with regard to the abortion procedure and were subsequently administered the first dose, mifepristone (200 mg), in the clinic. All women were counselled on contraception according to clinical standard procedure in respective study site. Additionally, the doctor and woman agreed on the location of misoprostol administration together; this was not randomized, but was based on the woman’s preference and the doctor’s discretion. Subsequently, the women who had been identified by the doctor as being clinically eligible for the study met with a research assistant, who explained the study and obtained informed consent. Women who consented were randomly allocated to either return to the clinic for follow-up by a doctor (in-clinic follow-up group), or to assess their abortion outcome at home using an LSPT and a pictorial instruction sheet, and subsequently be followed-up by a research assistant over the telephone or at home (home-assessment group). Research assistants recorded the women’s socio-demographic profile and reproductive history, including previous use of contraception. Scheduled follow-up took place at 10–15 days after mifepristone administration. Women were informed that they could return to the clinic in case of any perceived complications, however, the women in the home-assessment group were advised to not return to the clinic for the purpose of contraception before their scheduled follow-up. This stipulation was made because interim visits would interfere with the home-assessment intervention, unless they were returning to the clinic due to complications or adverse events. The study sites were instructed to provide contraceptive counselling to all women, and to provide a suitable method, when applicable, on day 1 and day 3 (for the women who returned to the clinic for misoprostol administration (*n* = 333)) of the medical abortion. The women who returned to the clinic for misoprostol administration on day three met a doctor or a nurse and stayed in the clinic for 1–4 h depending on the study site and the distance to the woman’s home. On this day, the care provider completed the research questionnaire and recorded whether or not a contraceptive method was provided and, if so, which method was provided. However, no specific intervention or training in contraceptive counselling for the purpose of the study was given and when implementing and piloting the intervention we realised that providers at the study sites were reluctant to provide injectables already on day 1 or 3 due to fear of side-effects and increased bleeding post-abortion. Most providers preferred to provide the method at the time of follow-up, regardless of our attempts to encourage them to provide the method earlier. A maximum of three follow-up attempts were made within 1 month after the abortion, and women not found were considered lost to scheduled follow-up. However, these women were contacted retrospectively to confirm their abortion outcome.

A doctor or a midwife assessed women in the clinic follow-up group at follow-up, 10–15 days after mifepristone administration and provided contraception upon request. All women who returned to the clinic for follow-up were reimbursed for their travel expenses. The women in the home-assessment group were followed-up by a research assistant over the telephone or at home when the woman did not have a phone of her own, commonly the case among rural residents. Women were asked about their LSPT result and pregnancy symptoms. Additionally, the research assistant asked all women questions relating to contraceptive preference, use and intention, as well as about their abortion experience.

At the 2-week follow-up, women recruited to the study in the urban sites, and who owned a phone of their own, were asked to participate in the 3-month follow-up interview. Women in the rural sites were not considered for the 3-month cross-sectional survey due to their lack of mobile phones and because it was not considered feasible or appropriate to make another home-visit. Three months after their abortion, the same research assistant who met the women at 2 weeks telephoned the women who had agreed to a follow-up appointment. A maximum of three attempts were made before the women were considered lost to the 3-month follow-up. Questions regarding contraceptive use, method of initiation and time of initiation were asked. To facilitate a method for the women to remember their time of contraceptive initiation we asked whether contraception was initiated: immediately on day 1of the abortion; after misoprostol on day 3; at the time of the follow-up (2 weeks); at the time of the following menstruation (around 4 weeks); recently, which refers to 1 month previous to the 3-month follow-up (8 weeks); just now (12 weeks); or not at all. We also asked whether the woman had had any pregnancies or abortions in the last 3 months. Standardized questionnaires were used throughout the study.

### Definitions

The study sites offered copper-IUD, 3-month injection, oral pill and condom, which are referred to as modern contraception for the purpose of this study. In the results section, the reversible contraceptive methods are grouped into two groups: the injectable and copper-IUD as one group, and the oral pill and condom as another group. This is an attempt to categorise the methods according to: methods with a more ‘long-acting and user-independent nature’, although the injection is not considered a LARC by the WHO; and methods with a more ‘short-acting and user-dependent nature’. Additionally, in contrast to IUD and injectables, women who reported initiation of contraceptive pills or condoms at the 2-week follow-up, may or may not actually use these methods after administration, especially with regard to condom use. Contraceptive use was defined as current use or initiation at 2 weeks and 3 months. Contraceptive initiation refers to the time when women started using their contraception. Additionally, we asked questions regarding; contraceptive method of preference, and the intention to initiate the method preferred at 2 weeks or the reasons for not choosing a method. Women could select several methods of preference, however for the purpose of analysis of method preference and contraceptive counselling (Fig. 4), we chose to organise methods of preference accordingly: women, who preferred either injectable or IUD have been reported as preferring injectable or IUD regardless of any additional preferences. Women, who reported to prefer oral pill only responded oral pill or condom; women, who reported to prefer sterilization only responded sterilization or condom; and women, who reported to prefer condom responded no other method but condom. Intention to initiate was divided into two categories: ‘actual plan’, defined as today, this week, after recovered from abortion, and after next menses; and ‘no actual plan’, defined as when my husband moves back from the city, after next holiday, and sometime next year.

### Study outcomes and measures

The primary outcome of this study was women’s use of modern contraception post-abortion, compared between study groups, and measured at 2 weeks and at 3 months after the medical abortion. Due to the small sample size of women in the 3-month follow-up, these data were used to indicate a trend of overall contraceptive use and continuation. The contraceptive preference, choice and usage patterns at 2 weeks in the sub-set of women allowed us to compare patterns seen among the same women in the 3-month follow-up, and in that way validate the 3-month data considering the small sample size at the follow-up. Moreover, it allowed us to compare the contraceptive patterns found in the sub-set of women with the patterns in the total study population at 2 weeks and hence indicate a trend of overall contraceptive use at 3 months, while being aware of the socio-economic differences between the sub-sample population and the whole study population. Secondary outcomes included: women’s preferred contraceptive method; whether women had initiated their preferred method at the time of follow-up (2 weeks and 3 months); and if not, whether their intentions to initiate were according to an ‘actual plan’ or ‘no actual plan’ (as defined above). Moreover, associations between contraceptive use at 2 weeks and 3 months; and women’s socio-demographic and reproductive background; contraceptive counselling; time of provision of the contraceptive method; contraceptive intention at 2 weeks; and abortion experience in terms of satisfaction, were assessed.

### Randomisation and masking

Block randomisation, using blocks of six, was used and a computerised random number generator (Random Allocation Software 2.0) generated the randomisation list [18]. The staff who generated the list and prepared the opaque sealed envelopes used for randomisation were not involved in data collection. Details of the randomisation procedure are recorded in the study protocol [23].

### Statistical methods and analysis

All statistical calculations were made using SPSS (version 20) and R (version 3.0.3). Descriptive statistics are presented for all variables. Categorical variables are compared using *χ* ^2^-test or Fisher’s exact test when appropriate. Continuous data are presented as mean (range). A *p*-value below 0.05 and a 95 % confidence interval (95 % CI), presented as ±, demonstrate significant differences. Odds ratios (ORs) were derived using logistic regression with various explanatory variables. Where related ORs were significant, adjusted odds ratios (AORs) were derived with multivariate logistic regression. Use of contraception over time was illustrated by conducting Kaplan-Meier survival analysis where a log-rank test established significance.

Analyses were carried out for evaluable subjects (ES): all women with a successful scheduled follow-up at 2 weeks, regardless of whether their abortion was complete or whether they had taken the full course of the medical abortion or not. To compensate for difference in loss to scheduled follow-up between the study groups, the outcome of whether a woman had initiated contraception was analysed using intention to treat (ITT) analysis, including all women who were randomized to one of the study groups, assuming that none of the women who were lost to scheduled follow-up had initiated a method at 2 weeks, except where data for contraceptive provision on day three were available. All women who had a successful 3-month follow-up were included in the sub-set analysis.

The study was developed and coordinated by Karolinska Institutet, and Uppsala University, Sweden, and Action Research and Training for Health (ARTH), Udaipur, India. The trial was registered at Clinicaltrials.gov: NCT01827995. The Institutional Ethics Committees at ARTH, and the Health Ministry’s Steering Committee of Government of India approved the study.

## Results

**Fig. 1:**
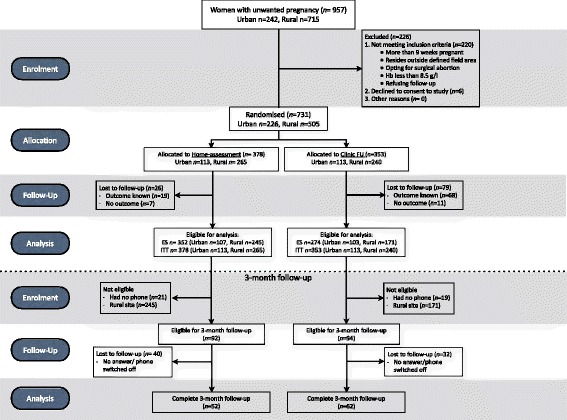
Study flow diagram. Flow diagram of the RCT showing enrolment, allocation to home-assessment (*n* = 378) or clinic follow-up (*n* = 353) group, the 2-week follow-up and analysis followed by the 3-month follow-up and analysis (*n* = 114)

A total of 957 women sought abortions and were screened for study eligibility during the study recruitment (April 2013 – June 2014); of these, 731 consented to participate and were randomized to either the in-clinic follow-up group (*n* = 353) or the home-assessment group (*n* = 378), and were included in the ITT analysis. In total, 626 women had a scheduled follow-up at 2 weeks and were included in ES analysis, and 114 women were successfully telephoned at 3 months, all of whom were included in the ES population, except one woman who was lost to scheduled follow-up at 2 weeks. The last follow-up was conducted in August 2014. The flow of patients is visualized in Fig. 1 and the participant profile is summarized in Table 1.

**Table 1:** Women’s socio-demographic and reproductive profile stratified by group allocation and 3-month follow-up

	ES population (*n* = 626)			3-month FU* Total *n* = 114
	Clinic FU *n* = 274	Home-assessment *n* = 352	Total *n* = 626	
Median age, years (range)	27 (18–48)	27 (18–46)	27 (18–48)	27 (18–43)
Residency, *n* (%)				
- Urban	92 (34)	94 (27)	186 (30)	101 (89)
- Rural	182 (66)	258 (73)	440 (70)	13 (11)
Belong to SC/ST Caste, *n* (%)	141 (51)	200 (57)	341 (55)	24 (21)
Level of Education, *n* (%)				
- No formal	124 (45)	202 (57)	326 (52)	10 (9)
- Primary (1–3 years)	51 (19)	49 (14)	100 (16)	11 (10)
- Secondary (4–10 years)	61 (22)	57 (16)	118 (19)	45 (39)
- Higher (>10 years)	38 (14)	44 (13)	82 (13)	48 (42)
Ownership of phone				
- Woman herself	130 (47)	155 (44)	285 (46)	114 (100)
- Husband	100 (36)	133 (38)	233 (37)	
- No/others	44 (16)	64 (18)	108 (17)	
Primigravida, *n* (%)	10 (4)	18 (5)	28 (5)	7 (6)
One or more living girls^a^	184 (70)	238 (71)	422 (71)	66 (62)
One or more living boys^a^	220 (84)	276 (83)	496 (83)	79 (74)
Median gestational age, weeks (range)	6.6 (5–9)	6.6 (5–9)	6.6 (5–9)	6.1 (5–9)
- Gestational age in weeks, *n* (%)				
< 6 weeks	51 (19)	59 (17)	110 (18)	27 (24)
6–7	150 (55)	197 (56)	347 (55)	73 (64)
> 7 weeks	73 (27)	96 (27)	169 (27)	14 (12)
Prior elective abortion, *n* (%)	106 (39)	106 (30)	212 (34)	55 (48)
- Medical^b^	79 (75)	85 (80)	164 (77)	46 (84)
- Surgical^b^	30 (28)	26 (25)	56 (26)	18 (33)
Home administration of misoprostol, *n* (%)	134 (49)	156 (44)	290 (46)	67 (59)
Ever-used modern contraception, *n* (%)	111 (41)	107 (30)	218 (35)	78 (68)

*The women in the 3-month FU are included in the ES population

^a^Presented as percentage of women with children (*n* = 264 in clinic FU and *n* = 336 in home-assessment)

^b^Presented as percentage of women with prior elective abortions (*n* = 106 in clinic FU and *n* = 106 in home-assessment)

There are no significant differences between the study groups within the ES population

There were no socio-demographic background differences between the women included in analysis and the women who were lost to follow-up (data of women lost to follow-up is not shown)

### Contraceptive use at 2 weeks and 3 months compared between study groups (*n* = 114)

**Fig. 2:**
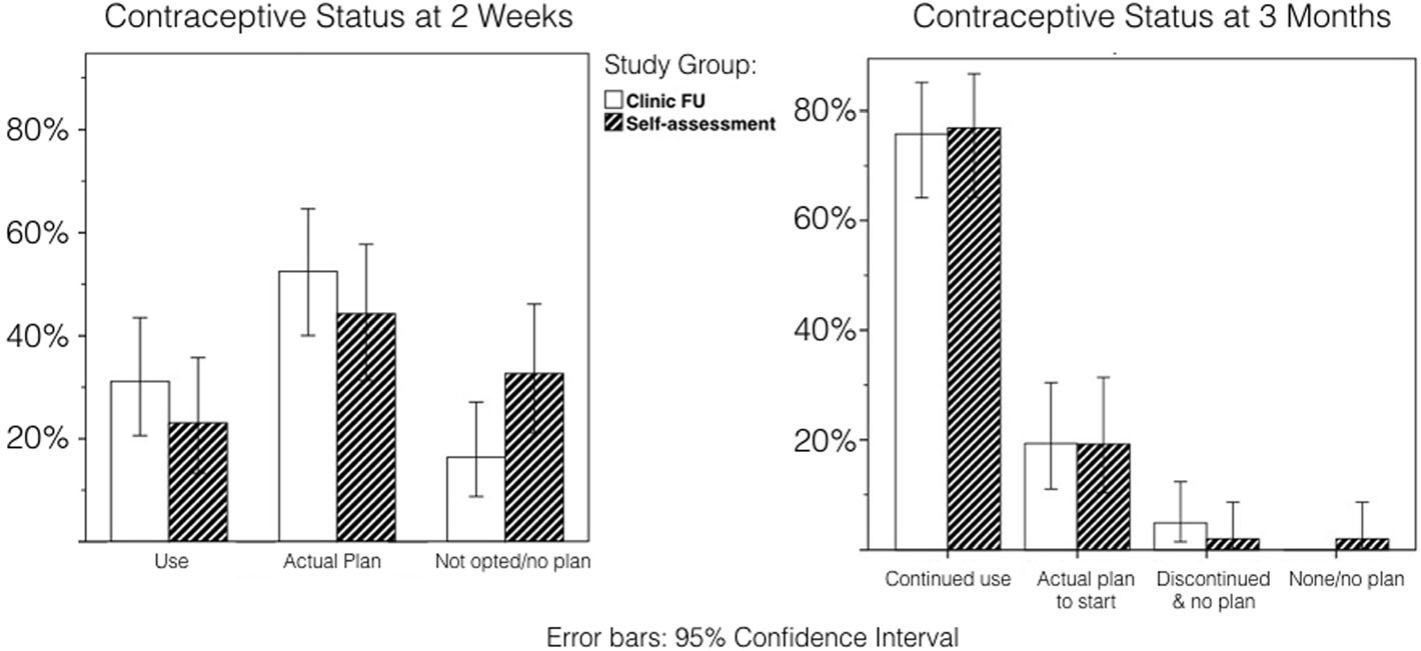
Comparing Contraceptive Status at 2 weeks and 3 months among the sub-set of women (*n* = 114). Left side: Women’s contraceptive status at 2 weeks: contraceptive use, whether has an ‘actual plan’ to use contraception, whether opted for a preferred method. Women were included if followed-up at 3 months (*n* = 114). Right side: Women’s contraceptive status at 3 months: contraceptive use and continuation, whether planning to start, discontinuation, and no plan to use contraception (*n* = 114)

**Fig. 3:**
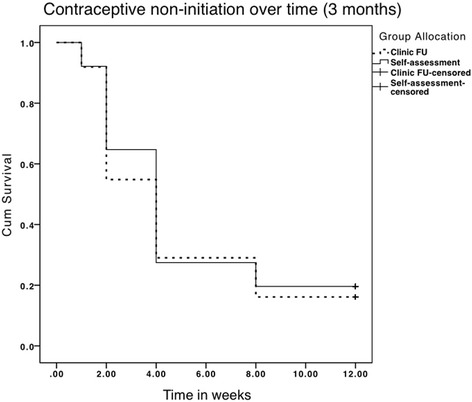
Contraceptive use over time (*n* = 114). Kaplan-Meier Survival Curve of contraceptive use over 3 months (12 weeks) as reported by the women where 2-weeks was at follow-up, 4 weeks represent after next menses, 8 weeks 1 month prior to 3-month follow-up, and 12 weeks refer to around the time of the phone-call. 1 event = contraception initiated. Stratified by treatment group (*n* = 114)

Most women (83 ± 6.9 %) had adopted and were still using (76 ± 7.8 %) a contraceptive method at 3 months post medical abortion, regardless of study group allocation (Fig. 2). There was no significant difference in contraceptive use between the study groups at the 2-week follow-up among the sub-set of women; however, there was a clear trend of increased use in the in-clinic follow-up group (Fig. 2). While women in the in-clinic follow-up group (*n* = 62) were most likely to initiate contraception at follow-up (62 ± 12 %), women in the home-assessment group (*n* = 52) were most likely to initiate contraception after next menstruation (60 ± 13 %). The trend of contraception initiation over time was analyzed with Kaplan-Meier Survival Analysis, illustrated in Fig. 3, where an event is defined as contraceptive initiation and the curve shows the non-initiation of contraception. Methods initiated at 3 months varied. Condom or oral pill were the methods that were mostly used, with no difference between study groups. However, 21 % (*n* = 11) of women in the in-clinic follow-up group had initiated the injectable or the copper-IUD compared with 10 % (*n* = 4) of the women in the home-assessment group. Three women had undergone sterilization (data not shown).

### Contraceptive use at 2 weeks compared between study groups (*n* = 626)

We analyzed contraceptive use at 2 weeks post-abortion in the ES population of the RCT (*n* = 626) (Table 2). Most women (89 %) intended to start a specific contraceptive method with no difference between study groups. Of those, 81 % opted for the oral pill, the 3-month injection or the copper-IUD, with no difference between the study groups. Overall, 33 % of ES population had initiated a method at scheduled follow-up. However, the question was only asked to women who had chosen a method of preference at follow-up (*n* = 556), and among these, 37 % had initiated a method, with a significant difference between study groups (clinic follow-up group 53 ± 6.2 %, and home-assessment group 25 ± 4.8 %) (Table 2). The ITT analysis (*n* = 731), where ‘no contraceptive use’ was imputed for women who were lost to scheduled follow-up and who did not initiate contraception on day one or three, reveals a smaller, yet significant difference between the study groups in terms of contraceptive use at 2 weeks (38 ± 3.5 % clinic follow-up group and 20 ± 2.9 % home-assessment group). Among the women who had an intention to initiate a specific method, but who had not yet initiated it at scheduled follow-up (*n* = 348), most (85 %) had an ‘actual plan’ to initiate, with no difference between study groups. For the women who did not wish to use any method (*n* = 67), reasons such as fear of side-effects (27 %), not cohabiting with their partner (20 %), not being aware of suitable methods (20 %) or lack of family support (14 %) were given (data not shown).

**Table 2:** Women’s contraceptive use and intention at 2-week follow-up (*n* = 626)

	Clinic FU *n* (% ± 95 % CI)	Home-assessment *n* (% ± 95 % CI)	Total *n* (% ± 95 % CI)	*p*-value
Preferred method for initiation chosen (*n* = 626)				
Yes	250 (92 ± 3.2)	306 (87 ± 3.5)	556 (89 ± 2.44)	0.059
No	22 (8 ± 3.2)	45 (13 ± 3.5)	67 (11 ± 2.44)	
Method initiated at 2 weeks (*n* = 556)				
Yes	133 (53 ± 6.2)	75 (25 ± 4.8)	208 (37 ± 4.0)	<0.001
No	117 (47 ± 6.2)	231 (76 ± 4.8)	348 (63 ± 4.0)	
Actual plan to initiate method at 2 weeks (*n* = 348)				
Yes	104 (89 ± 5.7)	192 (83 ± 4.8)	296 (85 ± 3.7)	0.154
No	13 (11 ± 5.7)	39 (17 ± 4.8)	52 (15 ± 3.7)	

Significant differences are indicated by *p* < 0.050. Missing values were excluded from analysis. Percentages are presented as column percentages. Actual plan to initiate was defined as: ‘after next menstruation’, ‘within 1 week’, ‘when recovered from abortion’

Among the women in the ES population who reported a preferred method (*n* = 556), significantly more women opted for condom in the clinic follow-up group (23 %) compared with the home-assessment group (16 %) (*p* = 0.025). Fewer women in the clinic follow-up group opted for the 3-month injection (44 %) compared with the home-assessment group (52 %) (*p* = 0.090). Only a small proportion of women (4 %) desired sterilization. Among all women who reported contraceptive use (*n* = 207), 52 % used the injectable (*n* = 95) or a copper-IUD (*n* = 13) with no difference found between the study groups (Table 3).

**Table 3:** Contraceptive method at 2 weeks stratified by study group: preferred and initiated

	Clinic FU	Home-assessment	Total	*p*-value
Method preferred (*n* = 556)				
Condom	58 (23 %)	48 (16 %)	106 (19 %)	0.025
Oral pill	61 (24 %)	62 (20 %)	123 (22 %)	0.242
Copper-IUD	30 (12 %)	37 (12 %)	67 (12 %)	0.974
3-month injection	111 (44 %)	158 (52 %)	269 (48 %)	0.090
Sterilization	6 (2 %)	18 (6 %)	24 (4 %)	0.044
Method initiated (*n* = 207)				
IUD or injectable^a^	73 (55 %)	35 (47 %)	108 (52 %)	0.295
Oral Pill/Condom	60 (45 %)	39 (53 %)	99 /48 %)	0.295

Method preferred refers to women who chose a method for initiation at 2 weeks however did not necessarily initiate it at 2 weeks (*n* = 556). While method initiated refers to the women who reported to have started at 2 weeks (*n* = 207)

^a^IUD refers to Copper Intra-uterine device (IUD) and injectable to the 3-month injection

### The influence of women’s background characteristics on contraceptive use

Social group (caste), educational attainment, and place of residence did not influence contraceptive use at 2 weeks or 3 months (data not shown). However, age was influential and more women aged 25–29 years had initiated a method at 2 weeks (38 %), compared with women aged 20–24 and 30–35 years (28 and 26 %). Few women (2 %) below 20 or above 35 had initiated a method at 2 weeks (data not shown).

When adjusting for number of children (non-significant), women were more likely (AOR 1.8, 95 % CI 1.0–3.0) to report contraception use at 2 weeks if they had a boy. Women with the intention to limit number of children were more likely (OR 1.8 95 % CI 1.1–3.0) to have chosen a method of preference, compared with women who wanted to space. Women’s previous use of contraception (*n* = 218) positively influenced contraception use at 2 weeks (OR 1.4 95 % CI 1.0–2.0) and having had a previous induced abortion (*n* = 212) was associated with having an ‘actual plan’ to initiate contraception as reported at the 2-week follow-up (OR 2.8 95 % CI: 1.3–5.9) (data not shown).

### Influential factors on contraceptive use

**Fig. 4:**
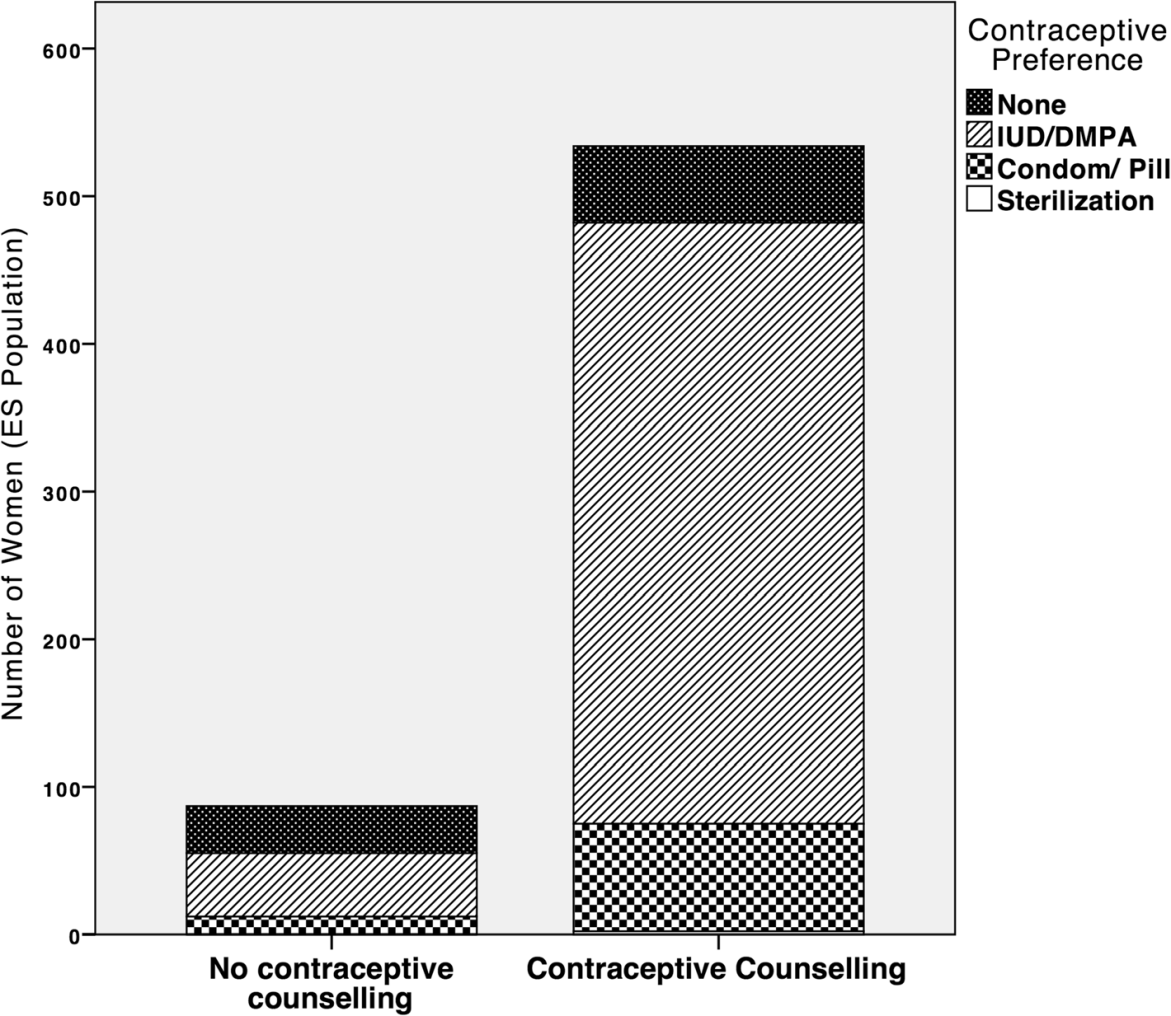
Contraceptive Counseling and Preference of Method (*n* = 626). Type of contraceptive method preferred by the woman stratified by whether the woman reported to have received contraceptive counselling nor not. Methods are categorized into injectable and IUD, Condom and Oral Pill, and sterilization. Women could choose to prefer more than one method, however for the purpose of analysis only one preferred method per woman is illustrated

Most women (86 ± 2.7 %) reported having received contraceptive counseling during their abortion, with no significant difference between study groups (90 ± 3.6 % in the clinic follow-up group and 83 ± 3.9 % in the home-assessment group). Ninety percent of the women, who received contraceptive counseling preferred a modern contraceptive method and 76 % preferred the injectable or copper-IUD, 14 % preferred the oral pill or condom and two women preferred sterilization (Fig. 4). These results were significantly (*p* < 0.001) different to those for women who reported no contraceptive counseling, where only 63 % preferred any method, of these, 49 % preferred either the injectable or copper-IUD and 14 % preferred the oral pill or condom (Fig. 4). Contraceptive counseling was associated with three times higher odds (AOR 3.4 95 % CI 1.5–7.8) of having reported contraception use at 2 weeks, when adjusted for contraceptive method provision on day three. Moreover, women who were provided with a contraceptive method on day three of the abortion were four times (AOR 3.7 95 % CI 2.0–6.9) more likely to have initiated contraception at 2 weeks compared with the women who did not receive or initiate a contraceptive method on day three when adjusting for women’s reported contraceptive counseling (data not shown). Moreover, being satisfied with the abortion positively influenced whether women chose a method at 2 weeks or not (OR 3.5 95 % CI 1.4–8.7) and having chosen a method and reporting an ‘actual plan’ to initiate at 2 weeks was associated with a six-times increased odds of contraception use at 3 months (OR 5.9 95 % CI: 1.2–28.4) (data not shown).

## Discussion

Our study shows that there is no difference in contraceptive use 3 months post-abortion among women whose abortion outcome was assessed in-clinic compared with women who assessed their abortion at home, bearing in mind that women in the 3-month follow-up mainly resided in urban areas where health care is easier to access. The post-abortion contraceptive use at 3 months seen in our study corresponded with results from previous studies in similar settings [11]. Interestingly, at the 2 week follow-up we found a higher overall contraceptive use (33 %) after medical abortion than was previously seen in similar settings in India (9 %) [10, 11]. This was true regardless of study group allocation and in spite of that most of the study participants in the RCT belonged to disadvantaged social groups and where the majority of participants resided in rural areas. However, there was a difference in method use between study groups. A larger proportion of women (53 %) in the clinic follow-up group had initiated contraception in comparison with women in the home-assessment group (25 %). A Nordic RCT investigating home-assessment post-medical abortion indicated higher (55 %) overall use 2 weeks post-abortion than was seen in our study, and with no difference between the study groups [19]. In spite of the Nordic setting being different from our study setting, our findings from the 3-month follow-up and the Nordic RCT suggest that with comprehensive contraceptive counseling and available contraception, women may be motivated to initiate contraception post-abortion irrespective of location of outcome assessment in both high- and low-resource settings. Even though the 3-month follow-up was carried out among women, who were mostly residing in urban areas, it is important to highlight that most study participants belong to underprivileged social groups with a relatively low socio-economic status, with women recruited at the rural sites having the lowest status. Our study shows that the contraceptive intentions reported by women at 2 weeks in the sub-set of women and in the total study population are comparable. Moreover we show that social group, educational attainment and residency do not affect women’s contraceptive use. Based on this we suggest that the observed 3-month contraceptive use in the sub-set of women can be used to indicate an overall trend of contraceptive use at 3 months in general, bearing in mind that the women in the sub-set had a slightly higher socio-economic status and resided in urban areas where health care is easier to access. The slightly postponed use of contraception among the home-assessment women is similar to what was previously observed when comparing contraceptive use after medical versus surgical abortion [11]. Thus, the delay in our study may be an effect of sub-optimal contraceptive counseling and provision rather than the course of abortion, whether home- or clinic-based.

To optimize contraceptive provision in medical abortion we need to understand what factors contribute to contraception use in low-resource settings. The WHO safe abortion guidelines state that contraceptive counseling must be provided on day one of abortion, and that most contraceptive methods are feasible to initiate on day one or three of the medical abortion [2]. Our study enforces this statement by showing that contraceptive counseling on day one, provision of a method on day three, and having an actual plan to initiate on day 14 were associated with contraceptive use at 2 weeks and 3 months. In line with this finding, previous research shows that having a reproductive intention, with regard to spacing or limiting childbearing, motivates women to adopt contraception [18]. We believe that effective provision of intra-abortion contraception could circumvent much of the decreased use at 2 weeks that was seen in the home-assessment group in our study, because there were no differences in the women’s intentions to initiate contraception between the study groups.

Women in our study preferred reversible or barrier methods and rarely desired sterilization. This finding is in sharp contrast to the widespread use of female sterilization, representing two-thirds of contraceptive use in India [24]. Interestingly, the 3-month injection was the overall preferred method, especially among the rural residents, supporting a trend documented in recent studies from similar settings [6, 12], and arguing for the inclusion of the 3-month injection under the NRHM family planning initiative. Additionally, hormonal IUDs and implants should be included as contraceptive options, given their long-acting nature, to avoid discontinuation soon after initiation [25, 26]. Such methods may also be preferable for the women because their use could be kept secret from other family and community members, and hence increases the feasibility of using the method [9]. Similarly, it has been argued that women in situations of dependence are more likely to avoid jeopardizing their social relationships, in this case by using contraception, to avoid the risk of rejection and loss of support [27]. Another study showed that Nepali women who received condom and oral pill post-abortion were more likely to discontinue within 12 months [28], supporting the importance of focusing on long-acting reversible contraceptive methods. In the total study population, women in the in-clinic follow-up group were more likely to prefer condoms at 2 weeks. This finding may be attributed to the patient-provider power-dynamic, putting women in a vulnerable position where they do not want to disobey the provider who is offering contraception, combined with women’s lack of autonomy resulting in their unlikeliness to act on their own preference [29]. This indicates the importance of empowering women to choose their method of preference from a range of different methods as well as encouraging them to return to the clinic if they want to discontinue or switch methods. In line with previous studies [6, 30, 31], our study showed that a woman’s age, existing family size, and whether the woman had any sons influenced contraceptive use. This supports the persisting misconceptions and fear of infertility related to contraceptive use. However, with the observed changes in the childbearing norms in India, young women living in rural areas have new opportunities to negotiate their reproduction [9]. The young women’s self-identified need for effective contraception, and their wish to space between and limit the number of children, provides the health care system with an excellent opportunity to motivate and increase women’s adoption of modern contraception [9].

Our study emphasized the importance of effectively offering contraception with a focus on the 3-month injection, the copper-IUD and other LARCs, intra-abortion. Early initiation of contraception is crucial to avoid repeat unintended pregnancies. Research shows a rapid return of ovulation post-abortion and most women ovulate before returned menses, however, ovulation may occur as early as 10 days post-abortion [32]. Hence, to wait to initiate contraception beyond 10 days post-abortion puts women at risk of unintended pregnancies, however, increasingly so if contraceptive provision is postponed until after the next menses [32]. Most LARCs and the 3-month injection, can be initiated on the same day as misoprostol, or after the confirmed expulsion of the pregnancy [33, 34]. Moreover, early insertion of hormonal IUD reduces the number of days of heavy bleeding post-abortion [34], which is beneficial in settings where anemia is common [35]. In line with these findings, Nepali women who adopted LARC post-abortion were less likely to discontinue or experience unplanned pregnancies within 1 year [6, 28]. Unfortunately, copper-IUDs or injectables were rarely provided on day three of the abortion in our study, primarily due to lack of routine and providers’ reluctance to do so. This can partly explain the lesser use of these methods in the home-assessment group at 2 weeks, and 3 months. However, the difference in use of IUD and injectables at 3 months must be further investigated to understand how to facilitate women’s initiation of long-acting methods, if they assess their abortion outcome at home, particularly because more women in the home-assessment group had expressed their preference towards the IUD or the injectable at the 2-week follow-up compared with women in the in-clinic follow-up group. Moreover, providers’ attitudes to and knowledge of contraceptive services are crucial for the provision of contraception. A study of Indian medical students identified that they possessed poor knowledge and had misconceptions with regard to modern contraception [36]. Hence, more research and clear clinical guidelines are required to support health care providers in their provision of contraceptive methods in medical abortion, especially with regard to early initiation of LARCs and the 3-month injection. In-service training of contraception in abortion care is crucial and should be combined with training in ‘simplified early medical abortion’ as this has proven to be effective and acceptable in low-resource settings [18, 20, 37].

To our knowledge, no previous RCT has investigated contraceptive use post-simplified-follow-up after medical abortion in a low-resource setting. However, there is a need for research on rural women’s contraceptive use and continuation over time as well as a more extensive study on urban women’s contraceptive use over time due to the small sample size in our study. Moreover, studies should preferably investigate contraceptive use over a longer time period than 3 months post-abortion. Another limitation of our study was that women in the home-assessment group could not return to the clinic for contraceptive initiation before their scheduled follow-up. This may have resulted in fewer women reporting contraceptive use by the time of their follow-up appointment, and may have affected the difference in contraceptive use between study groups. Moreover, women in the clinic follow-up group were reimbursed for their travel costs to decrease the dropout rate [23]. This encouraged women to return to the clinic and offered a better opportunity for contraception provision than is commonly seen [21]. These scenarios could have been circumvented by the provision of contraception on day one or three; however, this was not the clinical practice in most of the study sites. Finally, the risk of recall bias must be taken into account. At the 2-week follow-up, women were asked whether they had received contraceptive counseling on day one, and whether or not they had initiated a method before the 2-week follow-up. One can argue that women who had initiated a method were more motivated to report that they had received counseling, however, judging from the data and that most women reported contraceptive counseling regardless of contraceptive use at 2-weeks, indicates the validity of our findings. Moreover, if necessary, the research assistant could carefully probe for information about different aspects of the contraceptive counseling session, such as whether the woman remembered that the doctor spoke of methods to avoid pregnancy, this was to help the woman remember the encounter. However, and more importantly, for the women who were followed-up after 3 months, we asked when they had initiated the method of contraception. To facilitate these responses, the research assistant referred to actual events, such as ‘at follow-up’, ‘after next menstruation’ etc., rather than asking for time intervals in weeks. These events were then translated into number of weeks for the purpose of the survival analysis.

## Conclusion

Simplified follow-up after early medical abortion will not change women’s opportunities to access contraception in a low-resource setting, if contraceptive services are provided as intra-abortion services as early as on day one. Women’s post-abortion contraceptive use at 3 months is unlikely to be affected by mode of follow-up after medical abortion, also in a low-resource setting. Allowing women the choice of follow-up may motivate the use of LARCs or the 3-month injection. However, to optimize contraceptive counseling and provision in abortion care, all women should be offered a range of evidence-based contraceptive methods as early as on day one of their medical abortion, regardless of age or parity.

## Abbreviations

AOR: Adjusted odds ratio
ARTH: Action Research and Training for Health
CI: Confidence interval
ES: Evaluable subjects
IUD: Intra-uterine device
LARC: Long-acting reversible contraception
LSPT: Low-sensitivity pregnancy test
NRHM: National Rural Health Mission
OR: Odds ratio
RCT: Randomised controlled trial
SC: Scheduled castes
ST: Scheduled tribes
WHO: World Health Organization

## References

[1] Cleland J, Conde-Agudelo A, Peterson H, Ross J, Tsui A (2012). Contraception and health. Lancet.

[2] World Health Organizaton (2012). Safe abortion: technical and policy guidance for health systems.

[3] Curtis C, Huber D, Moss-Knight T (2010). Postabortion family planning: addressing the cycle of repeat unintended pregnancy and abortion. Int Perspect Sex Reprod Health.

[4] Sedgh G, Hussain R (2014). Reasons for contraceptive nonuse among women having unmet need for contraception in developing countries. Stud Fam Plann.

[5] Ali MM, Cleland JG, Shah IH. Causes and consequences of contraceptive discontinuation: evidence from 60 demographic and health surveys. Geneva: World Health Organization (WHO). 2012.

[6] Padmadas SS, Lyons-Amos M, Thapa S (2014). Contraceptive behavior among women after abortion in Nepal. Int J Gynecol Obstet.

[7] Ministry of Health and Family Welfare. District Level Household and Facility survey, DLHS-3. Mumbai: International Institute for Population Sciences (Deemed University). 2008.

[8] Bellizzi S, Sobel HL, Obara H, Temmerman M (2015). Underuse of modern methods of contraception: underlying causes and consequent undesired pregnancies in 35 low- and middle-income countries. Hum Reprod.

[9] Paul M, Essén B, Sariola S, Iyengar S, Soni S, Allvin MK. Negotiating Collective and Individual Agency : A Qualitative Study of Young Women’s Reproductive Health in Rural India. Qual Health Res. 2015. [Epub ahead of print]. 10.1177/1049732315613038 PMC5302084 26531879

[10] Zavier AJF, Padmadas SS (2012). Postabortion contraceptive use and method continuation in India. Int J Gynecol Obstet.

[11] Kalyanwala S, Acharya R, Francis Zavier AJ (2012). Adoption and continuation of contraception following medical or surgical abortion in Bihar and Jharkhand, India. Int J Gynaecol Obstet.

[12] Jejeebhoy SJ, Zavier AJF (2012). Injectable contraceptives: Perspectives and experiences of women and health care providers in India.

[13] National Rural Health Mission, Ministry of Health and Family Welfare, Government of India (2010). Comprehensive abortion care - training and service delivery guidelines.

[14] Jejeebhoy S, Francis Zavier AJ, Acharya R, Kalyanwala S (2011). Increasing access to safe abortion in rural Rajasthan: outcomes of a comprehensive abortion care model.

[15] Stillman M, Frost JJ, Singh S, Moore AM, Kalyanwala S. Abortion in India : A Literature Review. New York: Guttmacher Institute. 2014.

[16] Fiala C, Gemzel-Danielsson K (2006). Review of medical abortion using mifepristone in combination with a prostaglandin analogue. Contraception.

[17] Von Hertzen H, Huong NTM, Piaggio G, Bayalag M, Cabezas E, Fang AH (2010). Misoprostol dose and route after mifepristone for early medical abortion: a randomised controlled noninferiority trial. BJOG An Int J Obstet Gynaecol.

[18] Iyengar K, Paul M, Iyengar SD, Klingberg-allvin M, Essén B, Bring J, Soni S, Gemzell-danielsson K (2015). Self-assessment of the outcome of early medical abortion versus clinic follow-up in India: a randomised, controlled, non-inferiority trial. Lancet Glob Heal.

[19] Oppegaard KS, Qvigstad E, Fiala C, Heikinheimo O, Benson L, Gemzell-Danielsson K (2014). Clinical follow-up compared with self-assessment of outcome after medical abortion: a multicentre, non-inferiority, randomised, controlled trial. Lancet.

[20] Paul M, Iyengar K, Essén B, Gemzell- Danielsson K, Iyengar SD, Bring J, et al. Acceptability of Home-Assessment Post Medical Abortion and Medical Abortion in a Low-Resource Setting in Rajasthan, India. Secondary Outcome Analysis of a Non-Inferiority Randomized Controlled Trial. PLoS ONE. 2015;10(9):e0133354. doi:10.1371/journal.pone.0133354. 10.1371/journal.pone.0133354 PMC4556554 26327217

[21] Grossman D, Ellertson C, Grimes DA, Walker D (2004). Routine follow-up visits after first-trimester induced abortion. Obstet Gynecol.

[22] Hopewell S, Clarke M, Moher D, Wager E, Middleton P, Altman DG, Schulz KF (2008). CONSORT for reporting randomised trials in journal and conference abstracts. Lancet.

[23] Paul M, Iyengar K, Iyengar S, Gemzell-Danielsson K, Essén B, Klingberg-Allvin M (2014). Simplified follow-up after medical abortion using a low-sensitivity urinary pregnancy test and a pictorial instruction sheet in Rajasthan, India - study protocol and intervention adaptation of a randomised control trial. BMC Womens Health.

[24] Int Inst Pop Sci. National Family Health Survey (NFHS-3), 2005-06, India: Key Findings. Mumbai: International Institute for Population Sciences (IIPS). 2007.

[25] Langston AM, Joslin-Roher SL, Westhoff CL (2014). Immediate postabortion access to IUDs, implants and DMPA reduces repeat pregnancy within 1 year in a New York City practice. Contraception.

[26] Barros Pereira I, Carvalho RM, Graça LM (2015). Intra-abortion contraception with etonogestrel subdermal implant. Eur J Obstet Gynecol Reprod Biol.

[27] Ouédraogo R, Sundby J (2014). Social determinants and access to induced abortion in Burkina Faso: from two case studies. Obstet Gynecol Int.

[28] Puri M, Henderson JT, Harper CC, Blum M, Joshi D, Rocca CH. Contraceptive discontinuation and pregnancy postabortion in Nepal: a longitudinal cohort study. Contraception. 2015;91(4):301–7. doi: 10.1016/j.contraception.2014.12.011. Epub 2014 Dec 30. 10.1016/j.contraception.2014.12.011 25553872

[29] Elul B (2011). Assessments of the importance of provider characteristics for abortion care: data from women in Rajasthan, India. Health Care Women Int.

[30] Ertopçu K, Inal MM, Ozelmas I (2005). Demographic analysis of post-abortive and interval-administered hormonal contraceptive methods. Eur J Contracept Reprod Health Care.

[31] Lauro D (2011). Abortion and contraceptive use in Sub-Saharan Africa: how women plan their families fertility control in sub-Saharan Africa. Afr J Reprod Health.

[32] Stoddard A, Eisenberg DL (2011). Controversies in family planning: timing of ovulation after abortion and the conundrum of postabortion intrauterine device insertion. Contraception.

[33] Sääv I, Stephansson O, Gemzell-Danielsson K (2012). Early versus delayed insertion of intrauterine contraception after medical abortion - a randomized controlled trial. PLoS One.

[34] Gemzell-Danielsson K, Kopp Kallner H, Faúndes A (2014). Contraception following abortion and the treatment of incomplete abortion. Int J Gynecol Obstet.

[35] Jain M, Modi J (2015). An audit of obstetric admissions to intensive care unit in a medical college hospital of central india: lessons in preventing maternal morbidity and mortality. Int J Reprod Contraception Obstet Gynecol.

[36] Hogmark S, Klingberg-Allvin M, Gemzell- Danielsson K, et al. Medical students’ knowledge, attitudes and perceptions towards contraceptive use and counselling: a cross- sectional survey in Maharashtra, India. BMJ Open. 2013;3:e003739. doi:10.1136/bmjopen-2013-003739. 10.1136/bmjopen-2013-003739 PMC3863118 24334156

[37] Ngoc NTN, Bracken H, Blum J, Nga NTB, Minh NH, van Nhang N, Lynd K, Winikoff B, Blumenthal PD (2014). Acceptability and feasibility of phone follow-up after early medical abortion in Vietnam: a randomized controlled trial. Obstet Gynecol.

